# Endothelial dysfunction determines vascular mechanisms of metastatic progression in breast cancer

**DOI:** 10.1007/s12672-026-04437-y

**Published:** 2026-01-17

**Authors:** Matluba Mirzaeva, Akbarjon Mirzayev, Bakhtiyar Iriskulov, Sevara B. Azimova, Shamsiddin Nizamkhodjaev, Akmal M. Asrorov

**Affiliations:** 1 Tashkent State Medical University, Tashkent, Uzbekistan; 2 Central Asian University, Tashkent, Uzbekistan; 3 Kimyo International University in Tashkent, Tashkent, Uzbekistan; 4 Zhongshan Institute for Drug Discovery, Shanghai Institute of Materia Medica, Zhongshan, China; 5 Alfraganus University, Tashkent, Uzbekistan

**Keywords:** Metastases, Breast cancer, Intravasation, Extravasation, Endothelial dysfunction, Arterial hypertension, Diabetes mellitus

## Abstract

Breast cancer (BC) is one of the most common types of cancer among women, leading to death. More than 90% of deaths from BC are due to hematogenous metastases. In the cellular and tissue levels, the aggressiveness of BC is linked with epithelial–mesenchymal transition, leading to enhanced cell motility and spread in the bloodstream, leading to tumor expansion. This review focuses on the vascular mechanisms that facilitate metastatic dissemination in BC, highlighting the influence of blood vessels, hemodynamic forces, and vascular microenvironmental properties on tumor cell spread. Particular emphasis is placed on the role of endothelial dysfunction within common comorbid conditions and how these alterations may increase metastatic propensity. In addition, we discussed whether improving endothelial health and effectively managing comorbidities could help reduce metastatic progression.

## Introduction

Breast cancer (BC) metastasis is one of the major problems in clinical oncology and the leading cause of death. When a favorable environment is created, tumor cells leave the primary niche and spread in the bloodstream to various organs, where they undergo uncontrolled proliferation, forming new tumor nodules. This hematogenous spread is the cause of approximately 90% of cancer-related deaths [1].

The process of metastasis involves very complex and multi-stage mechanisms, including local invasion, intravasation, circulation, extravasation, colonization, and tissue environment regulation [1, 2]. Migration of tumor cells is accompanied by processes such as remodeling of extracellular matrix (ECM), angiogenesis, epithelial–mesenchymal transition (EMT), and mesenchymal–epithelial transition (MET). All of these are regulated by active enzymes (MMP-9, MMP-2), cytokines (IL-6, TGF, VEGF), and other proteins, which are overexpressed by tumor cells or accumulated in the tumor microenvironment [2–4].

Several scholars and groups have proposed theories to explain the mechanisms of metastases. The most common of these was Steven Paget’s “Seed and soil” theory, published in the Lancet in 1889 [1, 5]. He proposed that the metastases develop when the cancer cells (seeds) and the microenvironment of the host organ (soil) match each other. *James Ewing* formulated the hypothesis that the vascularization of the host organ plays a crucial role in the development of metastases [5]. Over the past few decades, numerous scientists have suggested that proteolytic enzymes, the organotropism of cancer cells, and specialized host cells create a microenvironment that stimulates the colonization of cancer cells [1, 5]. So far, all stages of metastasis, the characteristics of tumor cells and host cells, their interaction, and the organotropism of cancer cells have been studied at the molecular level. The five main stages of metastasis are invasion, intravasation, circulation, extravasation, and colonization, three of which are associated with vascular factors. Although individual prognostic factors of BC metastasis have been studied in many studies, the relationship between systemic vascular concomitant diseases, such as hypertension, diabetes, and atherosclerosis, has not been well studied. This complicates understanding how endothelial dysfunction modulates the metastatic potential in pathological conditions and how this can serve as an integrative predictor in risk subpopulations. In this narrative review, we focused on the above questions and tried to discuss the relationship between them.

In this overview, we performed a literature search without data restrictions to investigate the most recent evidence from articles indexed in the PubMed and Scopus databases. Published papers in these databases were searched using combinations of keywords related to breast cancer metastasis, vascular biology, endothelial dysfunction, and comorbid conditions. Both in vitro and in vivo experimental studies, as well as clinical investigations, were examined to provide a comprehensive understanding of the vascular-dependent mechanisms underlying the hematogenous spread of BC and the impact of comorbid conditions associated with endothelial dysfunction.

## Vascular-dependent stages of cancer metastasis

The spread of a tumor throughout the body and the formation of secondary foci occur through several stages, primarily within the vascular system. This process occurs through complex molecular mechanisms arising both within the tumor tissue itself and in the vascular wall. This section focuses on the key vascular-related stages that constitute the metastatic cascade and discusses their underlying pathogenesis.

### Cancer angiogenesis

**Fig. 1:**
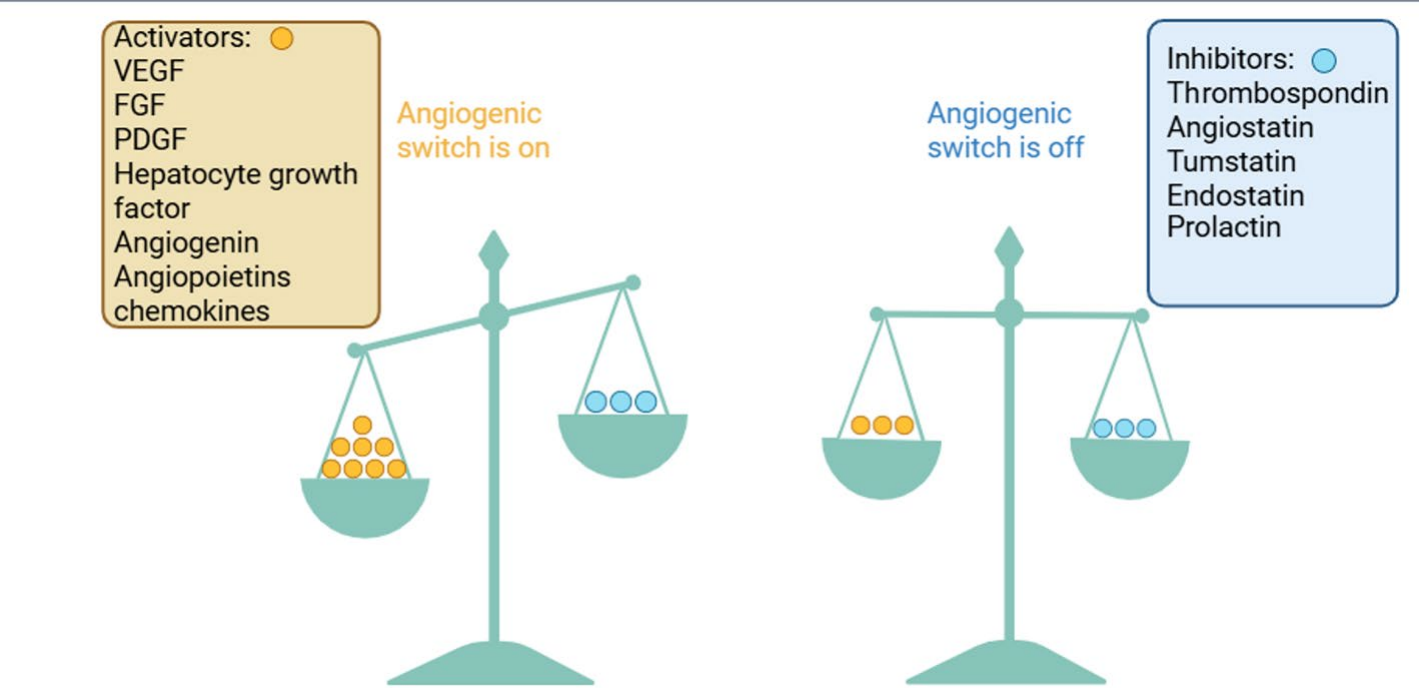
“Angiogenic switch” balance. Angiogenesis does not occur when activators and inhibitors are in balance. The figure describes the biological principle that angiogenesis is controlled by an equilibrium between activating and inhibiting signals. Due to the increase in activating substances in the tumor microenvironment, new vessels are formed. The figure was created with www.biorender.com (The figure was adapted from [6] with the permission of Wiley)

**Fig. 2:**
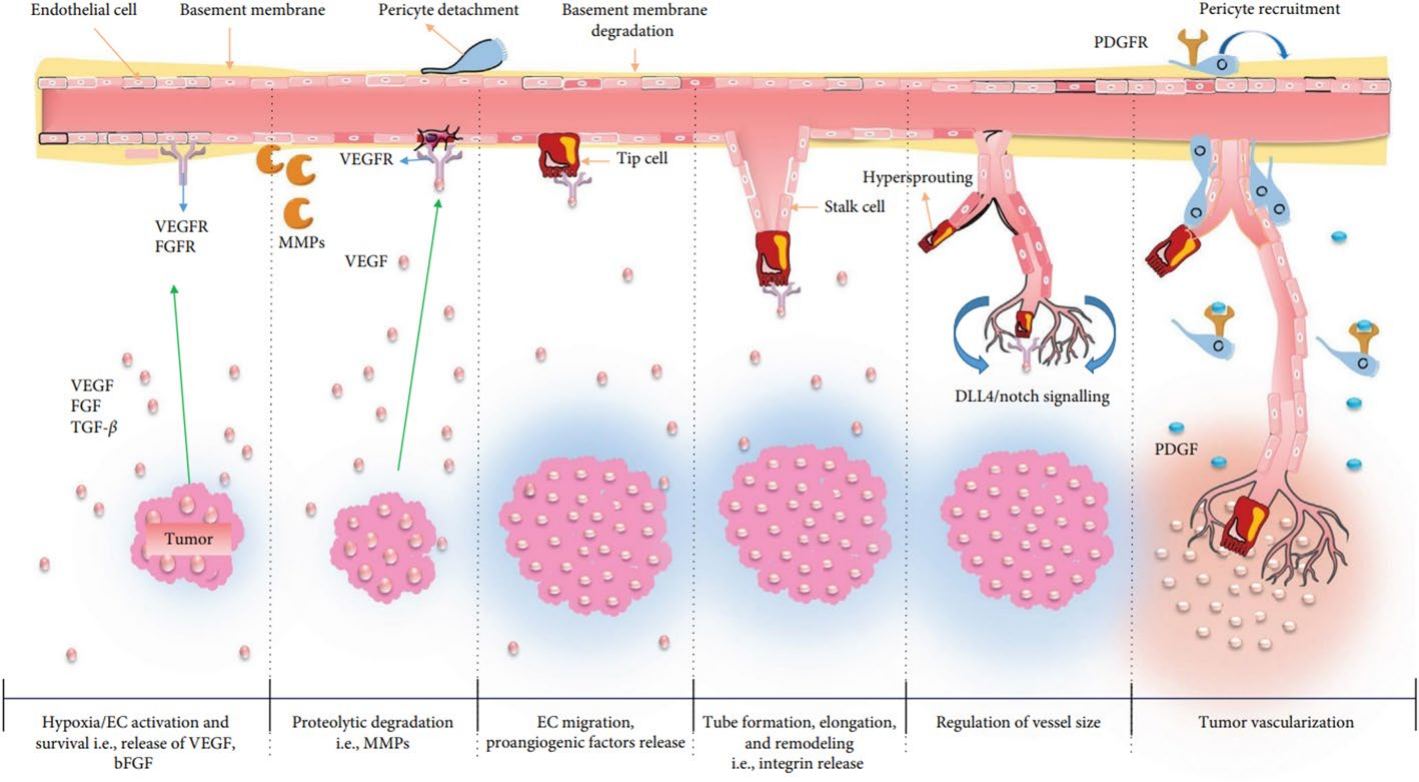
Mechanism of angiogenesis in cancer. Pro-angiogenic factors such as VEGF are released as a result of hypoxia observed in the tumor tissue, and the activity of protease expression increases. ECs migrate following the leading cells along the gradient of the angiogenic factor. PGDF stimulation promotes proliferation and the attachment of pericytes with reduced VEGF sensitivity. Blood supply stimulates further tumor growth (The Figure has been reused from [6] with the permission of Wiley)

Angiogenesis is one of the primary vascular processes in tumor development, which is regulated by vascular endothelial growth factor (VEGF). Pathological angiogenesis occurs as a response to various pathological processes in the body [6]. Angiogenesis in tumor tissues differs from that in normal tissues due to hypoxia and tumor microenvironment (TME) [7]. Several review papers were devoted to this topic. In this paper, we mainly focused on mechanisms related to blood vessels. In the complex TME, pro- and anti-angiogenic imbalance due to metabolic and mechanical stress, immune and genetic disorders activate various signaling pathways, which demonstrates the peculiarity of tumor angiogenesis [8–10]. The quantitative relationships of pro- and anti-angiogenic factors in one medium are referred to as the “angiogenic switch” (Fig. 1) [6, 11]. Tumors achieve high mitotic activation and proliferation by forming new blood vessels through various molecular and cellular mechanisms, thereby enhancing their oxygen and nutrient supply [12]. But hypoxia and ischemia in the TME affect endothelial Tip cells through various signaling pathways and pro-angiogenic factors [6]. Perivascular pericytes dissociate in parallel with the disintegration of endothelial cell (EC) junctions, which stimulates the proliferation of ECs. ECs following the filopodial Tip cells migrate to pro-angiogenic chemoattractant gradients in the TME; as a result, new blood vessels are formed (Fig. 2) [13]. An increased number of blood vessels in TME not only increases nutrients and oxygen supply but also stimulates hematogenous metastasis, as the newly formed blood vessels have high permeability, allowing tumor cells to enter the systemic circulation easily.

### Intravasation

Intravasation is a crucial stage of the metastatic cascade, in which tumor cells enter the bloodstream through the vessel wall. Tumor characteristics (such as tumor size and density) [14, 15], TME (e.g., hypoxia) [7, 16], and structural properties of local blood vessels (including microvessel density, permeability, and diameters) [15, 17] influence the intravasation. The interaction of all three mentioned factors jointly stimulates hematogenous metastases: during mitosis in large tumors, tumor cells cause the endothelium to bend, as a result of which intercellular connections between ECs are interrupted [15].

Invasive tumor cells undergo EMT, in which they modify from their epithelial behavior to mesenchymal and become more mobile, which allows them to invade surrounding tissues and spread to distant organs. Experimental findings showed that tumor cells subjected to EMT exhibit protrusive activity influenced by the Twist gene during basement membrane degradation. Morphometric and biochemical analyses demonstrated that invasive cells localize proteases within actin-rich invadopodia, which helps invasive cancer cells degrade the ECM components that make up the basement membrane. Subsequently, cancer cells develop pseudopodia, which play a crucial role in cell migration and are less invasive than invadopodia. Pseudopodia are larger than invadopodia, allowing the main body of the tumor cell to pass through the basement membrane and infiltrate the surrounding tissue and stroma [18].

In addition, several vascular conditions, like high vascular permeability or the lack of connections between ECs, are crucial for the transendothelial migration (TEM) of tumor cells. As the VEGF signal is activated, the tumor-derived VEGF loosens vascular endothelial-cadherin (VE-cadherin) and, as a result, vascular permeability increases [19]. On the other hand, the formation of new vessels requires both the degradation of the basal membrane surrounding the endothelium and the proteolysis of the collagen-rich ECM of the surrounding connective tissue. Several families of proteases, including metalloproteinases, cysteine cathepsins, serine proteases, and aminopeptidases, are responsible for matrix destruction during angiogenesis. Among them, matrix metalloproteinases (MMPs) are considered to play the leading role [20]. This degraded matrix can isolate various pro- and anti-angiogenic factors and change the balance of the “angiogenic switch.”

Tumor cells can easily enter the bloodstream through newly formed blood vessels since they are less specialized, sensitive to external factors, and have high permeability. The tumor cell migrates toward the blood vessel under the influence of the TME-derived chemokines, chemoattractants activated through MMPs that are released from the mediated degradation of the basal membrane, and the resulting chemical gradients. Cancer cells enter the bloodstream through the expanded endothelial space under VEGF influence. Tumor cells were shown to alter the activity of the cytoskeleton due to the sensitivity of the chemoattractant gradient [17].

**Fig. 3:**
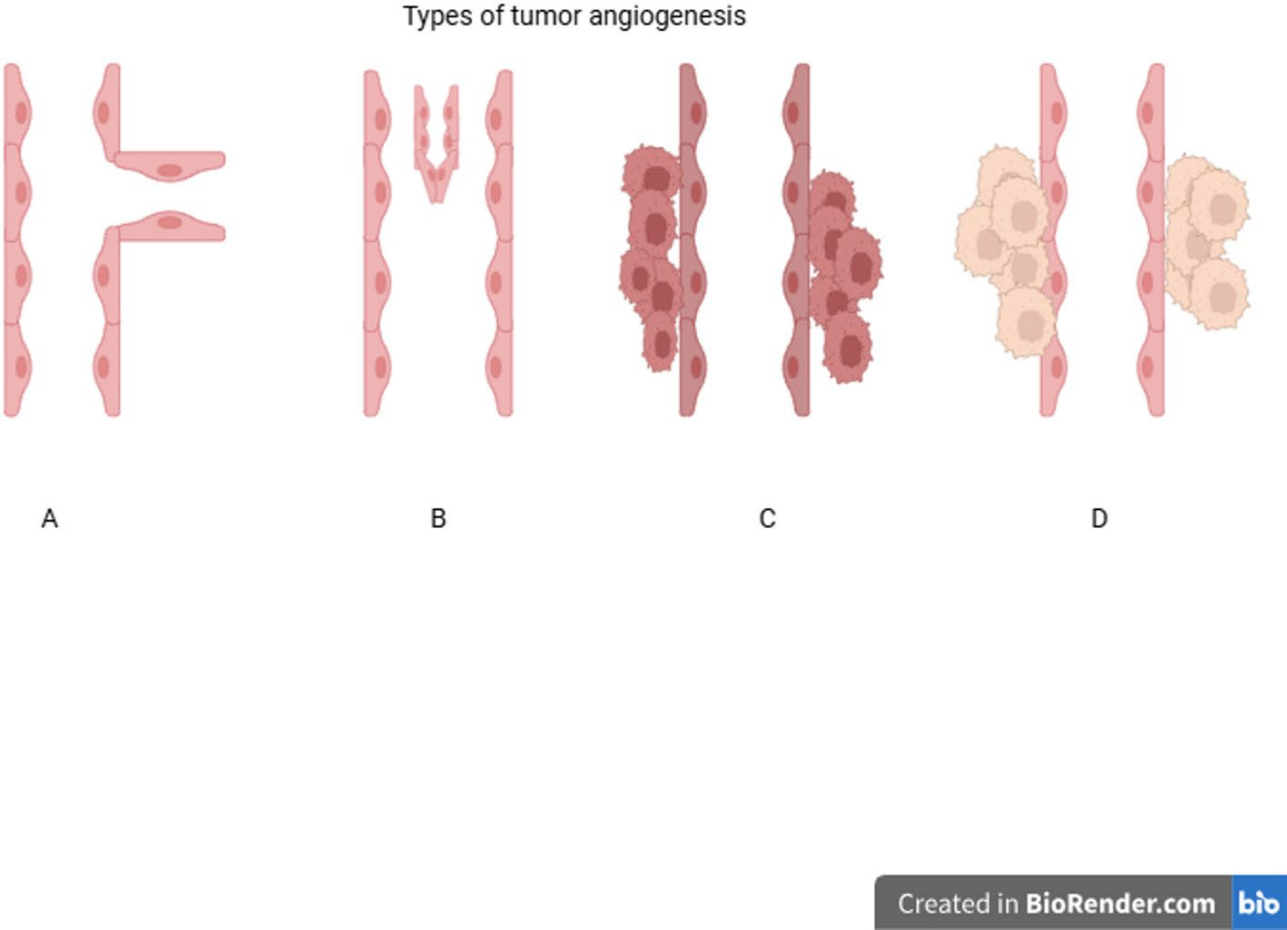
Mechanism of angiogenesis in cancer. Angiogenic dependent pathway: **A** Sprouting angiogenesis—new vessel formation through ECs proliferation and; **B** Intussusceptive angiogenesis—new vessels formation through invagination. Angiogenesis non-dependent pathway; **C** Vascular mimicry—tumor cells form vessel-like channels independent of endothelial cells, enabling blood perfusion through cancer-derived structures. **D** Vessel co-option—tumor cells, instead of inducing new angiogenesis, co-opt pre-existing host blood vessels and grow along their structure (The figure has been adapted from [21] with the permission of MDPI)

Wong et al. explained the relationship between tumor tissue and vascular endothelium and described intravasation as a model of tumor microvessels. The probability of tumor cells penetrate into blood vessels depends on the hardness of the ECM, the barrier function of the vessel wall, interstitial pressure, and matrix viscosity. When ECM has high hardness, it can mechanically affect the endothelium; therefore, it can be assessed as a factor that increases the risk of intravasation and metastasis in certain types of cancer, such as BC. If the endothelial intercellular connections are strong or the intercellular pressure is low, the endothelium can resist the damaging effect of the tumor cells. In cases of impaired endothelial integrity, when the adhesion of the tumor matrix is strong, it retains adherence to the endothelium and is prevented from being expelled by blood flow [15]. There is also evidence of the rearrangement of integrins and other adhesion molecules to facilitate the attachment of tumor cells to ECs [17].

### Vascular mimicry

The vascular mimicry (VM) is an unusual way of cancer blood supply of aggressive tumors, characterized by the ability of cancer cells to form vascular networks without the involvement of angiogenesis-dependent ECs. Figure 3C represents a factor contributing to intravasation and metastasis. Endothelial-like tumor cells secrete substances that contribute to the formation of the tubular structure (collagen IV and VI, proteoglycans, heparin sulfate, laminin, and tissue transglutaminase antigen 2), and subsequently, these tubules stabilize [12]. This condition is noted in aggressive types of cancer, including triple-negative breast cancer (TNBC) that has VM characteristics [22]. In the TNBC and HER2 (human epidermal growth factor receptor 2)-enriched types of BC, the frequency of metastasis is high, which, in many studies, is associated with the phenomenon of VM [23].

**Fig. 4:**
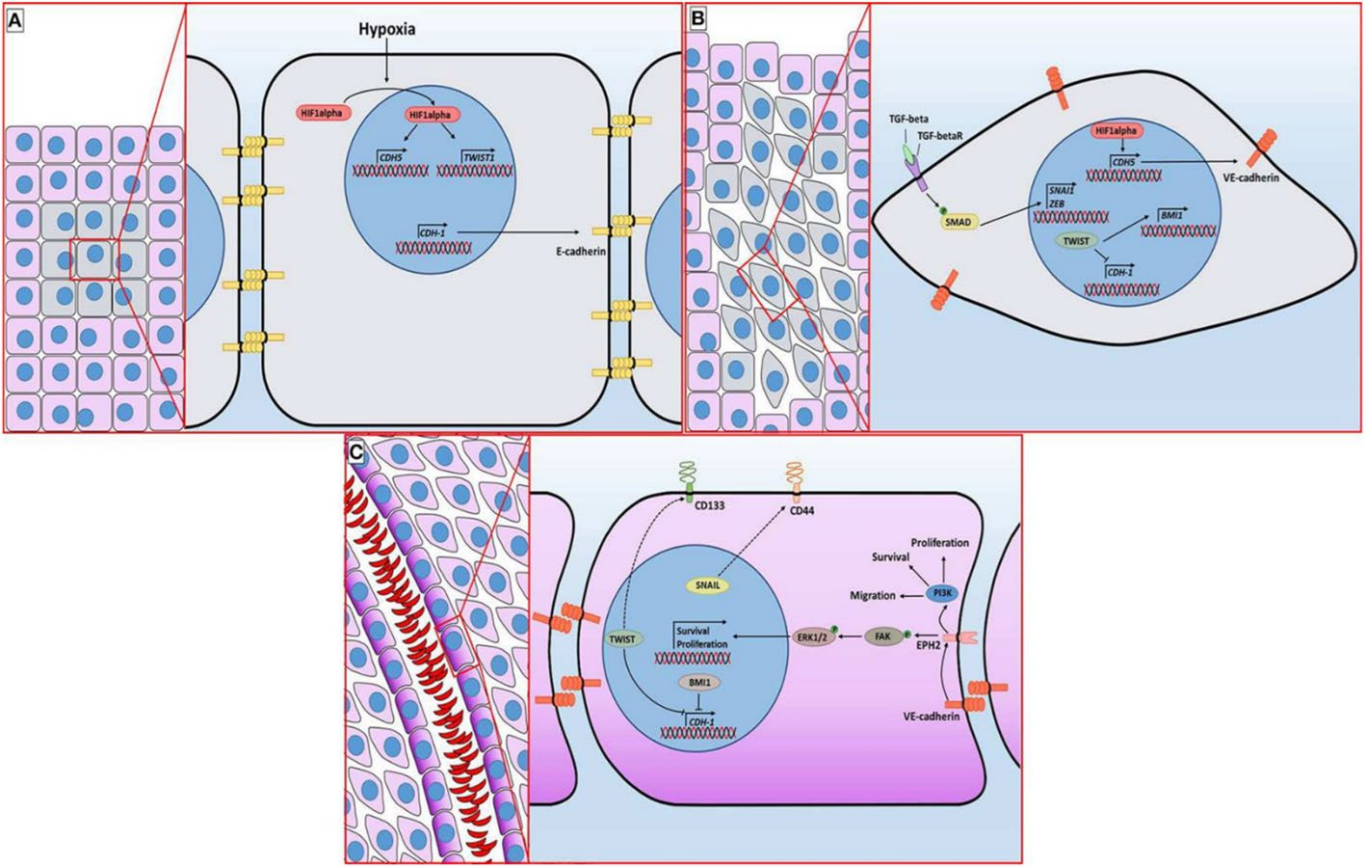
Molecular mechanism of vascular mimicry of BC. **A** The tumor initially displays an epithelial phenotype characterized by E-cadherin–mediated cell adhesion. **B** As hypoxia develops, HIF-1α (hypoxia inducible factor-1 alpha) is stabilized and activates Twist, driving epithelial–mesenchymal transition. Concurrent TGF-β signaling further promotes EMT, resulting in the replacement of E-cadherin with N-cadherin and acquisition of a mesenchymal phenotype. C - Tumor cells then express vascular markers such as EphA2 and CD44, align to form vessel-like channels, and permit blood flow through these structures—a phenomenon known as vasculogenic mimicry. This figure has been reused from [23] with permission of Frontiers

VM is the “internal pathway” of metastasis, and as a result of phenotypic changes in tumor cells, they acquire endothelial-like properties and create the possibility of self-release into the bloodstream. Under these conditions, tumor cells exhibit resistance to anti-angiogenic therapy(AAT). Anti-angiogenic drugs, while inhibiting vascular growth, increase the level of hypoxia, which causes the activation of Twist genes through HIF-1α (hypoxia inducible factor-1 alpha) and stimulates the VM process (Fig. 4). From a clinical point of view, VM can be a new target for treatment, as a causative means of metastasis, because of the absence of ECs in VM. Tumor cells can directly enter the bloodstream without losing the junction between ECs. At the same time, the presence of VM can be a potential biomarker for assessing the prognosis of the tumor [23, 24].

### Circulating tumor cells’ survival strategies in the bloodstream

**Fig. 5:**
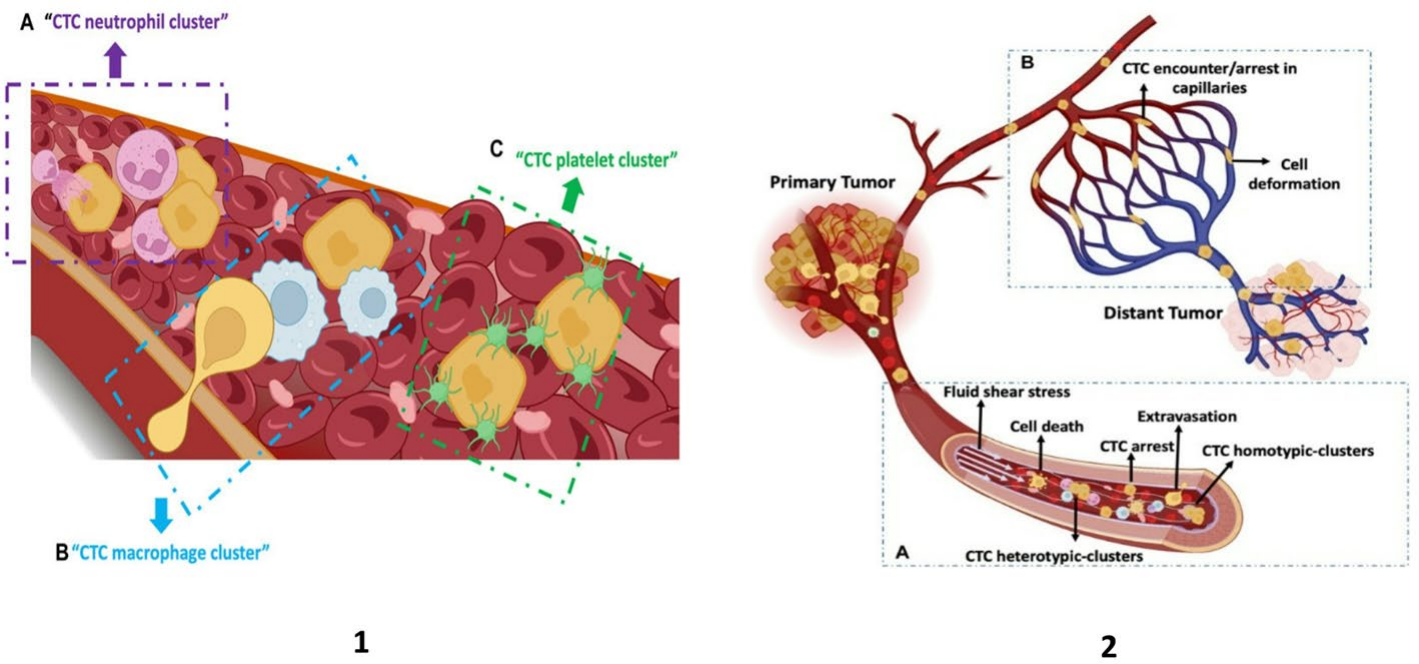
CTCs in the blood circulation. (1) Heterogeneous clusters of CTC with neutrophils (**A**), macrophages (**B**), and platelets (**C**). (2) CTC circulating system: homo- and heterotypic CTC cluster I circulation and their experience of/in blood fluid shear stress (**A**), CTC arrest in capillary and their deformation under biomechanical forces (**B**). Overall, the figure describes the formation of the distant tumor from the primary tumor (The figure has been reused from [28] with the permission of Frontiers)

Tumor cells that enter the bloodstream after intravasation are termed circulating tumor cells (CTCs) and are an essential part of the metastatic cascade. It is estimated that in cancer patients, there is one CTC per 1–10 million leukocytes [25]. This means that for the tumor to grow secondarily, it must survive attacks from white blood cells, which are millions of times more abundant in the bloodstream. Most CTCs are eliminated in the bloodstream due to strong hemodynamic forces, immune surveillance, metabolic or oxidative stress, and anoikis. However, a subset of CTCs can survive by activating specific adaptive mechanisms that allow them to overcome these resistance barriers [25, 26]. One of them is the formation of clusters of tumor cells with each other and with blood cells (Fig. 5), which has 20–100 times higher metastatic capacity than single cells [25–29]. Such clusters make CTCs resistant to fluid shear stress (FSS), tangential force of blood flow to the vessel wall, and other mechanical forces in the bloodstream. A high FSS in the arteries leads to a large number of CTC deaths. In the veins and capillaries, on the contrary, there is a low FSS, which ensures the arrest and extravasation of the CTC [30]. CTCs in a cluster state were found to be protected against mechanical deformation and apoptosis caused by this force. It should also be noted that even mechanical compression of CTCs in small capillaries reduces survival rates [28]. Studies conducted using the Zebrafish model showed that CTC clusters are liable to deformation to pass through the capillaries [31]. Resistance to such capillary pressure is also associated with EMT in cancer cells, which reduces cell stiffness and increases their ability to deform. This adaptation is supported by cytoskeletal restructuring, a phenomenon also observed in BC models [28]. Types of BC with a high frequency of metastasis are TNBC and HER2/enriched subtypes. D’Andrea et al. have shown that in these types of BC, CTCs have high deformation and clustering properties [21].

Heterogeneous clusters consisting of platelets or neutrophils protect against attacks by immune cells, including natural killer cells or adaptive immunity cells [29]. Moreover, strategies for CTC survival, such as avoiding the effects of anoikis and oxidative stress, and avoiding immunity, are covered in detail in several articles [21, 25–29, 31]. We did not elaborate on other mechanisms, since we mainly covered the strategy of survival in relation to the influence of vascular forces.

In metastatic BC, the detection of CTCs represents one of the strongest prognostic indicators, with ≥ 5 CTCs per 7.5 mL defining an aggressive disease phenotype and < 5 CTCs indicating a more indolent course [32]. Although CTCs are detected infrequently in early BC, even the presence of a single CTC (≥ 1 CTC/7.5 mL) is associated with markedly reduced distant metastasis–free survival(MFS) and overall survival(OS). Notably, the prognostic value of CTCs is independent of tumor subtype [33].

### Extravasation

**Fig. 6:**
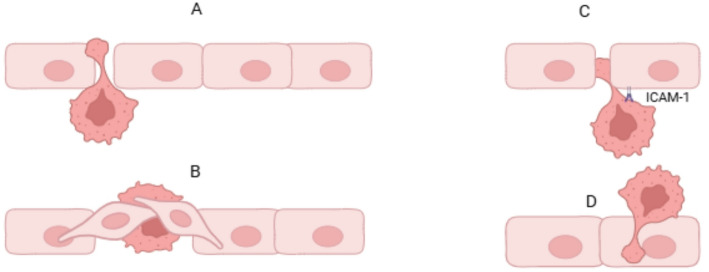
Extravasation types: **A** Active extravasation (diapedesis/protrusion): Cancer cells actively form protrusions that invade the endothelial barrier. Through cytoskeletal remodeling, they push into endothelial junctions and initiate their own passage into surrounding tissues. **B** Passive extravasation (angiopellosis): In this mechanism, cancer cells are extruded by endothelial remodeling rather than by their own motility. **C** Paracellular migration: Tumor cells migrate between endothelial cells by loosening intercellular junctions. **D** Transcellular migration: Cancer cells pass through individual endothelial cells, using transendothelial pores or channels. The figure was created with www.biorender.com

Extravasation is one of the critical steps of cancer metastases, which is defined by CTC exiting from the lumens of blood vessels to the new tissue—premetastatic niche (PMN). A tiny fraction (0.01%) of CTCs can extravasate and colonize, avoiding the influence of mechanical and biological factors in the bloodstream. Most of the CTCs may eventually transition to a dormant state or die in the new environment [26, 34]. As arrested in small capillaries, the CTCs first form a weak and occasional adhesion to the endothelium. Subsequently, this connection is strengthened, and TEM of tumor cells can be implemented. Several mechanical factors, including selectins, cadherins, integrins, and immunoglobulins, as well as blood flow velocity, are necessary for the adhesion of tumor cells to ECs [35, 36]. CTCs can extravasate from the blood flow in two ways: actively by diapedesis and passively by angiopellosis (Fig. 6). The first approach requires cancer cells to pass through the endothelial junction, and at the same time, only a CTC passes through the ECs barrier [35].

In the first Twist-induced pathway, the extravasation of tumor cells does not depend on integrin. Accordingly, in this case, the extravasation of tumor cells is not associated with vascular remodeling. The protrusive cell phenotype carries out extravasation with amoeboid movement of tumor cell migration, independent of integrin adhesions and ECM degradation [31]. TEM can occur in two ways. The paracellular pathway occurs due to the loss of connections between ECs and increased permeability. Inflammatory cytokines are also involved in this process [37]. E-selectin-mediated adhesion of cancer cells was found to increase their motility, while in EC, it increases endothelial permeability and contributes to extravasation [34]. The second pathway is the transcellular pathway, in which cancer cells penetrate ECs directly. Several experimental models have proven that tumor cells use invadopodia in this way. Through protease-rich invadopodia, the tumor cell degrades the ECM and performs TEM [35].

In angiopellosis, ECs remodel around a single CTCs or cluster, leading to their extravasation from the vascular lumen. The vessel wall thickens and leads to an increase in the number of ECs junctions in the arrest zone, which increases the number of potential sites for tumor cell extravasation [31, 35, 38].

Because TNBC is the BC subtype with the highest metastatic propensity, its underlying mechanisms have been studied more extensively than in other subtypes, and numerous in vivo and ex vivo experiments have demonstrated that these processes are tightly regulated by the behavior of ECs and blood flow dynamics [39–41].

Factors secreted from brain ECs activate the EGFR-DOCK4-RAC1 pathway in TNBC cells, reorganizing their cytoskeleton and enhancing deformability, thereby accelerating their passage through the capillary endothelium. Consequently, TNBC cells actively cross the complex architecture of brain microvessels and exhibit increased metastatic potential within the cerebral microenvironment [39]. In BC, the organs in which metastases most frequently occur exert a defining influence on the rate and direction of tumor-cell extravasation through their vascular architecture. Ex vivo two-photon microscopy demonstrated that hepatic sinusoids permitted 56% of tumor cells to extravasate within 24 h, whereas the more restrictive pulmonary endothelium allowed only 22%, confirming that metastatic organotropism is tightly governed by the permeability characteristics of the host organ vasculature [41].

### Organ selectivity

Organ selectivity is another significant factor playing a role in extravasation. It is dependent on blood flow, vascular diameter, and molecular composition of the endothelial surface in the pre-metastatic organ [34]. Chemotaxis can also occur in the direction of tumor cells due to the quantity of attractants released from the PMN. In studies, CXCR4-CXCL12 signaling was found to be associated with bone metastases, while other chemokine-receptor pairs are associated with liver and lung metastases [28]. However, these three organs with the highest metastases have high metabolic activity and are well supplied with blood [42]. The PMN is formed before extravasation: mediators of inflammation (IL-6, TNF-α, S100 proteins) and immunosuppressive cells accumulate, creating a special environment close to the endothelium. PMN signals (e.g., via VEGF and MMPs) increase ECs permeability. CTCs can also support the “arrest” phase by restructuring collagen and fibronectin. In these areas, the endothelial barrier weakens, and the conditions support the release of CTCs. With the maturation of PMN, CTCs contribute to infiltration from blood vessels and attraction of tumor cells to the niche, which actively stimulates the formation of metastases [42].

The role of adhesive molecules in the endothelium during extravasation is another crucial point deserving attention. Many articles have indicated the involvement of various adhesive proteins in the metastatic cascade [43–46]. CTCs in the bloodstream must adhere to the endothelium inside the target organs before extravasation. The CTCs in the bloodstream initially weakly bind to the endothelium in a short time to counteract stress and prevent leakage. Weak binding occurs between selectins on the endothelial surface and CD44 and MUC1 in CTCs. At the beginning of the CTCs rolling on the endothelial surface, a weak attachment transitions into a strong adhesion. It is known that the binding of integrins (α5β1 and α4β1) in cancer cells with endothelial adhesive proteins (VCAM-1 and ICAM-1) provides this strong adhesion. The aforementioned adhesion molecules play an essential role in metastasis [43, 45]. The in vitro model, in which adhesion between tumor cells and brain endothelium under FSS was studied, demonstrated high levels of MUC1 and VCAM1, which are associated with brain metastases of BC from selected tumor cells [43]. CTCs, capable of binding to endothelial adhesion molecule-1 or P-selectin, cannot enter ECs until the thickness of the glycocalyx layer is reduced [45]. This indicates that endothelial adhesion molecules (selectins, ICAM-1, VCAM-1) play a central role in the extravasation process of tumor cells. They ensure the transition of CTCs from initial weak binding to the endothelium to strong adhesion and contribute to the formation of a stable connection even under physiological flow conditions.

Therefore, drugs or antibodies that target these molecules can be a promising therapeutic direction for stopping the early stages of metastasis. Endothelial glycocalyx are other markers used to assess the probability of metastatic risk preservation.

### Tumor vessels co-option

Tumor vessel co-option is one of the tumor strategies that ensures survival, growth, and metastasis (Fig. 3D). Unlike angiogenesis, cancer cells use pre-existing host vessels rather than inducing new vascular growth. This process is less dependent on the VEGF signal pathway and causes resistance to anti-angiogenic drugs [21, 47]. It is a vital stage of tumor colonization in the metastatic niche. The absence of reliable molecular markers has limited the understanding of how this vascular takeover occurs. Some studies suggest that it may be driven by EMT-associated cellular changes and enhanced tumor–endothelial adhesion. Therefore, the emergence of vascular co-option in tumors is likely influenced by EMT-related factors and their downstream signaling pathways [48].

Vessel co-option is common in lung, hepatic, and brain metastases of BC [49]. By analyzing the features of histopathological and vascular growth characteristics of 164 cases of lung metastases, vessel co-option was often observed in metastases of BC [50]. According to the results, this non-angiogenic strategy was found to a certain extent in 91% of BC cases, in 98% of colorectal cancer cases, and in 62% of renal metastases. Additionally, vessel co-option was dominant in lung metastases of BC (72%), colorectal cancer (78.9%), and kidney cancer (38%). The hijacking of host vessels is found in all molecular subtypes of BC, but is less common in TNBC [50].

When comparing the blood vessels of primary tumors and brain metastases in non-small lung cancer, it was found that in metastatic tumors, there are 63% more mature vessels than in neoangiogenic ones. These findings support the possibility that metastatic brain tumors rely more on vascular co-option than angiogenesis [51]. It can be concluded that a deep understanding of vascular co-option mechanisms and targeting can provide new possibilities for preventing the development of metastatic nodes and increasing the effectiveness of treatment.

## The role of blood vessel condition in the metastatic process

The properties of blood vessels can either facilitate or, conversely, hinder hematogenous metastasis. Assessing these properties can be a predictor of MFS in cancer survivors. For example, the microvessel density (MVD) in the tumor tissue is currently one of the important prognostic indicators for several cancers, including BC [52, 53]. In this section, we will examine the normal and pathological features of the vascular system and their influence on the metastatic process.

### Microvessel density

MVD plays one of the most important roles among the factors contributing to intravasation. With all other factors being equal, the tumor tissue with higher vascular density has a higher tendency to metastasize [17]. Studies demonstrated that the involvement of extratumoral and tumor-associated macrophages can increase vascularization in the tumor tissue [54]. These macrophages also contribute to tumor intravasation through increased vascular permeability [15]. *Hansen and his group* found a correlation between metastasis and high vascular density in the primary tumor in patients with colorectal cancer [55].

In BC cases, the prognostic value of MVD has been widely studied, although findings vary across molecular subtypes [56, 57]. Assessment of CD34 antigen showed that MVD is elevated in progesterone receptor (PR)-positive tumors, and that the TNBC type exhibits lower MVD than other subtypes of BC [58]. However, another study indicates the positivity correlation of MVD with high tumor grade and the absence of hormone receptors [53]. The hormone-negative subtypes of BC (TNBC and HER2/neu-enriched) are aggressive due to their high angiogenic potential, which may not always be captured by endothelial markers, like CD34, due to VM.

In clinical practice, MVD is the most widely used method for assessing cancer angiogenic activity [53]. Angiogenesis is a well-recognized hallmark of distant metastases, as demonstrated by several research works. In BC patients, MVD markers (CD31/34) can be identified as prognostic markers prior to initiating anti-VEGF therapy. Elevated MVD levels can help predict the effectiveness of AAT, while also considering the assessed or monitored probability of VM development of these cancers.

### Vascular permeability

Endothelial barrier dysfunction leads to hyperpermeability, characterized by a lack of cell-cell junctions and enlarged endothelial openings, which is essential for the progression, metastasis, and therapeutic target of cancer [37]. Increased vascular permeability and a decrease in the peritoneal/myofibrillar cell covering of ECs ensure the spread and dissemination of tumor cells into the vessel [59]. Sznurkowska reported the role of TME factors, intrinsic and mechanical factors in intravasation. Vascular permeability induced by VEGF due to hypoxia is one of these factors [45, 60, 61]. Three types of interaction between tumor and ECs lead to vascular permeability: tumor cell-mediated, primary tumor cells’ secreting factor-mediated, and resident cell-mediated [45]. Glycocalyx, which covers the surface of ECs, is also an essential factor in permeability [37, 45]. Due to the presence of glycocalyx, substances released from the tumor cannot bind to receptors in the endothelium. The breakdown of glycocalyx also increases the outflow of cells (extravasation) [37, 62]. In TNBC, the breakdown of the endothelial glycocalyx weakens the vessel’s protective barrier, allowing tumor cells to stick more easily to the endothelium and pass through the vessel wall more quickly [40].

Due to endothelial hyperpermeability, prolonged leakage of plasma proteins, including fibrinogen, occurs, which contributes to the formation of an inflammatory microenvironment, leading to the formation of a PMN. In experimental models, the presence of CCL2-CCR2 signals in the lungs and the accumulation of fibrinogen increase the risk of metastases [37, 45].

Chronic inflammation supports vascular wall changes, emphasizing the need to use stabilizing or modulating permeability agents to reduce tumor development and increase drug effectiveness [37, 45, 63]. Considering the role of fibrinogen in the formation of PMN, reducing or blocking its effect can become a strategy for preventing metastasis.

### Vascular diameter

Blood vessel diameter plays a crucial role in tumor progression, intravasation, adhesion, and extravasation [36, 64]. The blood vessels in the tumor tissue are dilated, curved, and unevenly distributed; their diameters range from 10 to 120 μm [31, 36, 65]. Tumor-secreting factors like VEGF and fibroblast growth factor are considered to increase vascular density and diameter [17, 36]. In some cases, the diameter of the vessel reaches up to 200 μm [64]. MMP-9 and other proteases, secreted by stromal cells of the TME, also serve to enlarge the diameters of vessels. This creates conditions for intravasation [17, 36]. In primary tumors, a vessel diameter exceeding 100 μm usually ensures intravasation. The mechanism of intravasation, through reduced high permeability and shear stress in large-diameter vessels, was studied in liver metastases from colon cancer [31, 36]. Based on location in the tumor, vessels of large diameter are located in the center of the tumor, while vessels of small diameter are located on the periphery [31, 36, 65, 66]. An inverse correlation was found between tumor diameter and oxygen levels, and a direct correlation with tumor grade [36, 64, 66]. The development of necrosis and hypoxia in the tumor center and the large vessel diameter can also be explained by low oxygen levels. TEM is facilitated in large-diameter and uneven vessels in hypoxic or paranecrotic zones [31]. Large vessels with a size starting from 50 μm increase the risk of metastasis [31, 36].

Extravasation is most often observed in narrow-diameter capillaries. Due to the narrow-vessel diameter equaling 5–8 μm, CTCs, having 10–20 μm size, become self-clogged [31]. Since they do not flow in such vessels, clogged tumor cells bind with adhesive proteins. In wide vessels, due to high blood flow, the bloodstream carries away the CTCs before they can adhere [65]. High-resolution images in animal models show that cells across the endothelium in vessels are 5–14 μm wide, often against blood flow, and use β1-integrin adhesion [31].

In the experimental model, it was also confirmed that the brain metastasis of the BC model reduces blood flow in small-diameter vessels by compressing them through the tumor’s co-option strategy. Through co-option strategies, tumor cells can supply themselves with blood through existing peripheral blood vessels around them (Fig. 3D). Antiangiogenic drugs, such as bevacizumab or cediranib, reduce the diameter and permeability of high-diameter and uneven vessels of the tumor, blocking angiogenesis. However, due to the lack of effect of AAT on small vessels (< 10 μm), the tumor supplies itself with blood through small-diameter vessels located on the periphery through the “co-option” strategy, which causes drug resistance [36, 66]. Mathematical and intravital models indicate optimal treatment methods by combining sequential antiangiogenic and anti-cooperative agents focused on diameter-dependent flow and adhesion [31, 65, 66].

In summary, wider vessels enhance intravasation and survival in circulation, while narrow ones facilitate adhesion and extravasation [17, 36, 65]. Large diameter tumor vessels increase permeability, hypoxia, and secondary angiogenesis, as well as stimulate metastasis. When applying treatment strategies, it is essential to block angiogenesis in dilated vessels and prevent co-option in narrow peripheric vessels [36].

### Vessel volume and blood flow in the metastatic cascade

The analysis of human autopsy materials showed that metastasis in more than 40% of cases depends on blood flow and geometry [67]. CTCs that enter the bloodstream spread randomly along the flow. Rapid flow in large vessels ensures the spread of CTCs, whereas slow flow in small vessels is essential for their arrest and extravasation. The animal model showed that CTCs arrest in 80% of cases depends on hemodynamic forces [65]. As mentioned in the CTCs section, these two conditions can also cause decreased CTCs survival. Earlier studies showed that multiple metastases in organs such as the lungs and liver are directly related to blood flow mechanics [30, 67]. The effect of large blood flow and narrow capillaries on CTCs can, on the other hand, be linked to natural selection because only non-viable cancer cells die. In the human blood vessel model, BC metastatic cells in vessels with a diameter of 6 μm exhibit damage to the nucleus and DNA, rapid blood flow velocity, and cell deformation at high FSS. However, high expression of EMT genes is observed in these cells, which are more adaptable to conditions, more viable, and more invasive [30, 68]. Blood flow depends on vascular geometry and pressure. Laminar flow increases the reliability of CTCs, while turbulent flow damages them. Feather beds are mainly observed in capillary and vascular bifurcations [65, 69]. In studies, low-flow zones at the bifurcation of vessels are very favorable for the cessation and adhesion of CTCs [69].

Metastasis is 40–80% dependent on hemodynamic forces. Regulation of blood flow and velocity (e.g., with anticoagulants) may be a novel approach to reducing metastasis [69]. In Table 1, we present the role of some vessel types of metastases and metastatic organotropism.

**Table 1:** Role of different vessel types in BC metastases

Vessels types	Key characteristic	Role in metastasis	Organotropism’s significance in BC	Refs.
Newly formed vessels	Irregular, incomplete-walled, and leaky	Easy intravasation of BC cells	Prominent in aggressive BC subtypes; promotes metastasis to the lung and liver due to leaky vasculature	[17]
Capillaries	Very narrow lumen and slow blood flow	Arrest of the CTCs	Critical in the brain and lung metastases, where narrow capillaries trap CTCs	[28, 31]
Veins	High permeability, low pressure, slow blood flow	Convenient for extravasation	Important in the lungs and bone marrow, where more veins allow CTCs entry into tissues	[34]
Arteries	The thick wall, high blood pressure, and fast blood flow	Long-distance spread of CTCs	Less direct role in BC; may contribute to systematic spread	[28, 29]
Sinusoidal vessels	Wide lumen, slow flow, discontinuous endothelium	Easy arrest and suitable for CTC extravasation	Highly relevant for liver and bone metastasis, BC cells preferentially colonize bone via sinusoidal marrow vessels	[28, 34, 42]

Systemic vascular networks in the body, and various changes in them, are also crucial in the circulation of CTCs and extravasation stages of the metastatic process, as well as organotropism. The influence of vascular diseases on BC metastasis will be considered in the next section.

## Influence of diseases with endothelial dysfunction on extravasation and organotropism of BC metastases

Comorbid conditions with cancer can either hinder or facilitate the disease diagnosis process. In the vascular stages of metastasis, as discussed above, the spread of the disease throughout the body depends on various vascular factors. Therefore, diseases with vascular damage alongside BC can speed up metastasis. The effect can be especially significant during the stages of extravasation and colonization (PMN preparation, organotropism).

In approximately 65% of women with BC, at least one concomitant disease was diagnosed at the time of the initial diagnosis [70]. Among comorbidities, arterial hypertension (AH) is the most common, followed by cardiovascular disease (CVD) and diabetes mellitus (DM) [71]. According to the statistics, dyslipidemia (67.5%), hypertension (44.3%), obesity (18%), and diabetes (17.3%) frequently occur concomitantly with BC. Diseases of the respiratory, endocrine, excretory, and digestive systems constitute a small portion [70]. In particular, when BC presents with DM, the initial diagnosis is often made in the advanced stage [72]. Clinical studies have confirmed the influence of comorbidities on BC treatment outcomes and patient survival [73]. Although many studies have studied the relationship between the above-mentioned comorbid conditions and cancer development, there are very few sources covering the impact of these diseases on metastasis. In this section, we will review the mechanisms that stimulate metastasis in diseases with systemic vascular endothelial dysfunction and compare them with clinical research findings.

### Arterial hypertension

Various relationships between AH and BC were studied to clarify the role of AH in cancer development. The results of a large meta-analysis confirm a positive relationship between AH and postmenopausal BC [74]. The consequences of CVD show similar results in women with BC and surviving BC patients [75]. Jung et al. showed the presence of hypertension in 44% of age-related survival in patients with metastatic BC [76, 77]. Sutton et al. also indicated the possibility of AH development in BC survivors [78]. However, there is not enough clinical evidence on the influence of CVD or AH on BC metastasis. Above, we analyzed the role of various vascular parameters in the metastatic cascade (Sect. 2), and vascular damage in AH can also trigger metastasis.

BC and AH are explained by the common pathophysiological pathway associated with chronic inflammation of adipose tissue. In another work, hypertension was suggested to block apoptosis, which can increase the risk of BC [74]. Some other studies link the development of BC with the use of long-term and disorderly usage of antihypertensive drugs [79].

Several factors, like smoking, poor diet (salty or fatty diet, sugar), older age, and obesity, can contribute to endothelial dysfunction in AH [80]. Nitric oxide (NO) is a primary regulator of healthy blood vessels and a potent vasodilator, produced by ECs [81, 82]. It protects blood vessels from inflammation, thrombotic aggregation, and cellular proliferation [80, 83]. The above factors lead to a decrease in NO production or bioavailability, which contributes to impaired endothelial-dependent vascular dilatation [81]. Oxidative stress plays a significant role in endothelial dysfunction by reducing NO production. A pro-inflammatory and reactive oxygen species (ROS)-enriched microenvironment, high shear stress, and oscillatory flow enhanced the reduction in NO production by blocking endothelial NO synthase (eNOS). On the other hand, the renin-angiotensin-aldosterone system (RAAS) is activated, and Endothelin-1 and Angiotensin-II (Ang II) increase, which stimulates vascular constriction [81–84]. Chronic endothelial dysfunction can lead to structural changes in the vasculature [82]. Due to the limitation of the vessel’s expansion response to shear stress, the thickening and elasticity of the vessel walls decrease. Vascular remodeling leads to arterial stiffness due to decreased elastin and increased collagen in the ECM, and proliferation of smooth muscle cells [84]. As a result, the lumen of the vessels narrows, shear stress increases, vascular permeability increases in inflamed vessels, leading to the expression of adhesive molecules (ICAM, VCAM-1). Assessed by the metastatic process, all of the above changes may serve to arrest or extravasate the CTCs.

### Diabetes mellitus

In DM, high sugar levels are considered the main factor of endothelial dysfunction. High sugar intake can lead to vascular damage by causing stress, inflammation, and vasodilation disorders. Chronic hyperglycemia contributes to endothelial dysfunction through several interconnected pathways, including persistent inflammatory activation, reduced NO bioavailability, increased oxidative stress, accumulation of advanced glycation end-products, endoplasmic reticulum stress, and disruptions in angiogenic signaling. Together, these factors lead to impaired vascular function and an increased risk of vascular complications, such as atherosclerosis, hypertension, and microvascular diseases [80]. According to epidemiological studies, the occurrence of DM is observed in 12–16% of BC patients [85–87]. Evidence suggests DM is an independent predictor for the incidence and progression of BC, and has a high impact on the OS of patients, as well [88]. Especially in patients taking insulin, a cancer diagnosis is confirmed in the late stage (II-IV), and the risk of death is high [85, 89, 90]. In 5% of patients with DM, the fourth stage of the disease is detected during the initial diagnosis, and mortality is observed in 38.2% of metastatic BC patients with diabetes, which is higher than in patients without diabetes (30.1%) [91]. A population-based study reported that women with type 1 diabetes (T1D) have a 52% higher risk of developing BC [92]. In contrast, type 2 diabetes (T2D) is present as a comorbid condition in 8–20% of women diagnosed with BC. Moreover, T2D itself is associated with approximately a 15% increase in the risk of developing BC [89, 93].

The presence of DM has been associated with decreased MFS among BC survivors. There is a strong correlation between the presence of DM and metastasis, especially in subtypes with negative estrogen receptors (ER) [94]. Fundamental and clinical studies explain this connection through hyperglycemia, IR (insulin resistance), and insulin-dependent signaling pathways [88, 94–96]. Also, in T2D patients taking insulin, the development of metastases was faster, while in those taking metformin, the recurrence of disease and mortality decreased [89, 90, 93]. Park et al. reported that metformin use may be associated with a reduced incidence of ER-positive BC, while concurrently increasing the likelihood of ER-negative and TNBC subtypes [93]. Additionally, some evidence supports a potential therapeutic benefit of metformin in hormone-receptor–positive BCs; data regarding other tumor biological characteristics and the influence of different metabolic disturbances remain limited [97]. The overall effect of metformin is to improve endothelial function through the AMPK-NO pathway [90, 98], supporting its potential vasoprotective role in reducing metastatic spread and disease recurrence. So far, numerous clinical trials have shown that such a result has not been observed when metformin is included in BC therapy in patients without DM [90].

On the other hand, a recent study conducted by Rachman et al. reports no relationship between IR and metastasis in BC patients [99]. The divergence in research findings suggests that, while tumor growth and progression are influenced by insulin signaling, this factor alone does not fully account for the mechanisms of metastasis. In the context of DM, particularly under comorbid conditions, it is essential to consider additional vascular alterations—most notably microangiopathy and endothelial dysfunction—as potential contributors to metastatic dissemination.

### Obesity/Chronic inflammation

The effect of obesity on BC is explained by chronic inflammation associated with adipocytes [100]. In obesity, vascular permeability increases, and the expression of adhesive proteins is observed due to chronic inflammation. Moreover, active macrophages are the source of factors of the VEGF family, stimulating angiogenesis. CTCs in the bloodstream and reaching the target organ survive due to inflammatory mediators secreted by immune cells and begin colonization in the target organ [101, 102]. In experiments on mice, lung inflammation was induced. The formation of neutrophil extracellular traps (NET) in the process transformed dormant cancer cells into aggressive metastatic cells [103]. The awakening process of dormant cells is influenced by neutrophil elastase and MMPs released from NETs. Inflammation is important at each stage of tumor metastasis (Table 2) [104].

**Table 2:** Role of inflammation in the metastatic process

Steps of metastases	Mediators and cells	Mechanism	Refs.
EMT	TNF-α, TGF-β, IFN-α, IL-6(JAK-STAT3 pathway), IL-17, IL-23 (Wnt/β-catenin pathway), IL-1β	Acquire mesenchymal phenotype, increase motility, become more invasive, and enhance survival capacity	[104–106]
Intravasation	IL-6, TNF-α	EC junctions are disrupted, and the permeability of vessels increases	[17, 104]
CTC circulation	• EMT-associated tumor cells released immunosuppressive cytokines and increased mesenchymal molecules (vimentin) • Neutrophilic clusters of CTC • Thrombocytic clusters of CTC	Immune escape develops; cytotoxic T-cell responses are inhibited Leucocyte activation decreases Cancer cells are protected from natural killer cells’ attack	[21, 104]
Extravasation	Fibroblasts, neutrophils, and tumor-associated macrophages release chemokines and cytokines	Inflammatory PMN formation promotes CTC extravasation	[103, 104, 107]
Colonization	IL-6, TGF-β, HIF-1α, neutrophils’ leukotrien	Hypoxic microenvironment in the metastatic niche develops, and angiogenesis is induced	[104]

Clinical evidence suggests that obesity plays a crucial role in BC development and progression in pre-and postmenopausal women [108–112]. This phenomenon is explained by several interrelated factors, including metabolic dysfunction, chronic inflammation, hypercholesterolemia, and high estrogen levels [111, 113]. Meta-analyses showed that obesity affects the prognosis of BC differently depending on the stage. In early-stage patients, obesity significantly reduces the OS and disease-free survival (DFS), increases the risk of recurrence, and distant metastasis [112]. On the contrary, obesity in first diagnosed metastatic BC does not negatively affect the prognosis; in some cases, it can be neutral or even partially positive [114]. Olsson et al.. studied that central obesity (waist-to-hip ratio, WHR) is directly related to the risk of metastasis in BC [115]. In patients with high WHR, the 5-year risk of metastasis was 11.2%, almost 2 times higher than in patients with low WHR. This connection was strongly manifested in the racial (black women) and genomic subgroups (PAM50 ROR) of patients. This study also analyzed the issue of tumor organotropism, where obesity was associated with metastases to the lungs, liver, and bone, as well as multifocal metastases, and no significant differences were found in brain metastases [115]. These results indicate that obesity can affect not only overall survival but also the direction and load of metastasis. Therefore, managing the mechanisms associated with obesity for patients in the early stages is essential in preventing metastasis or disease recurrence. There is evidence about improving patient outcomes related to a weight loss strategy [116]. However, weight loss has increased the mortality rate in patients by three times and remains a bad prognostic sign of the disease. It is unknown what kind of change is associated with this increase in the number of deaths [117]. However, preclinical and clinical studies have confirmed that targeting chronic inflammation improves disease outcomes in cancer, including BC [118–121]. Non-steroidal anti-inflammatory drugs (NSAIDs), which suppress COX2 pathways, reduce tumor proliferation, invasion, and MMP activity, and increase the synthesis of anticancer proteins [121]. They also suppress angiogenesis by reducing PGE2 synthesis, thereby influencing the process of metastasis. Experimental models confirmed that NSAIDs suppress VEGF synthesis and reduce vascular density [119]. Scientific evidence shows that taking NSAIDs before and after treatment reduces the risk of distant metastasis of BC by 20–50% [118, 120]. However, the effect also depends on the type of cancer and the molecular subtype, and clinical benefit was more pronounced in hormone receptor-positive and HER2-enriched subtypes of BC [119].

Although numerous studies have demonstrated an association between obesity and an increased risk of metastasis, the available evidence remains incomplete [115, 122, 123]. In the early stages of disease, obesity has been linked to higher rates of recurrence and metastatic spread; however, its impact on prognosis once the metastatic stage is established is still controversial [116]. The mechanisms underlying organotropism in central obesity are poorly understood, and no clear biological pathways have yet been defined to explain obesity-related metastatic patterns. While current data suggest that controlling chronic inflammation may improve treatment outcomes, evidence from randomized clinical trials is still limited [118, 119, 121]. Consequently, the development of individualized strategies for managing cancer in the context of obesity is a critical area for future research.

### Atherosclerotic cardiovascular disease

Atherosclerotic cardiovascular disease (ACVD) is one of the conditions with structural vascular changes affecting metastasis. The results of one extensive cohort study, covering 21,654 people, lasting 6 years, confirmed the presence of a link between the development of cancer and ACVD in middle-aged patients. Besides, metastatic cancer was confirmed in 39% of patients with ACVD [124]. A major cohort study, conducted by Chinese scientists over 6 years, confirmed that patients with ACVD tend to have a higher incidence of cancer (especially digestive system cancer) as arterial stiffness increases [125]. This relationship can be explained by processes common to both states, such as inflammation, ROS, and metabolic disorders. The results of the study of BC and ACVD confirm the presence of a two-way relationship between the diseases [126–128]. A study by Scalia et al. on cell cultures of different BC lines confirmed that cancer cells oxidize LDL (oxLDL), and the resulting oxLDL causes the expression of adhesive molecules on the endothelial surface. oxLDL also caused the expression of LOX-1, CD36, and CD162 in monocytes. This condition increases monocyte adhesion to the endothelium, creating conditions that favor the development of atherosclerosis [126]. Recent clinical studies confirm the above experiment. In particular, Melson et al. emphasize that 2/3 of BC patients have a high risk of CVD and an increased risk of atherosclerosis due to insufficient treatment of these comorbid conditions, despite the recommendations [127].

TNF-α, IL-6, and IL-1β are important cytokines that link the pathogenesis of both diseases. TNF-α leads to the formation of atherosclerotic plaques by increasing vascular permeability and damaging the endothelium. It also leads to endothelial dysfunction by reducing NO bioavailability. Clinical studies have confirmed a strong correlation between TNF-α and distant metastasis of BC [129]. The effects of IL-6, IL-1β have also been proven in both directions, all of which emphasize that vascular damage is an important pathogenetic point in the progression and metastasis of BC, and indicate that therapies aimed at strengthening the vascular wall are a promising direction in preventing metastasis [129].

In 55% of BC patients, signs of subclinical atherosclerosis were detected during PET-CT examination [130]. Researchers note that calcifications in the thoracic arteries, recorded on mammography, are associated with the presence of risk factors for ACVD [128]. These findings emphasize the need for early detection of atherosclerosis using imaging methods and strengthening cardiovascular prevention.

Many studies have noted a high frequency of cancer confirmation at the metastatic stage in patients with ACVD at the time of diagnosis [124, 131]. However, there is no direct clinical evidence of how the degree of control of ACVD and its risk factors (hypertension, dyslipidemia) affects the development of metastasis. Therefore, the influence of cardiovascular diseases and uncontrolled risk factors on the risk of metastasis should be studied in special prospective studies.

There are very few studies on the association between ACVD and cancer progression and metastasis, and most of them focused on the possibility of ACVD presenting with metastatic cancer at the time of primary diagnosis. According to the clinical results, BC metastasis was observed in 8% of patients with ACVD [124]. New scientific studies monitoring primary diagnosed early-stage BC patients with ACVD can provide valuable findings in this direction.

## Conclusion and future direction

Endothelial dysfunction, increased vascular permeability, and structural remodeling of the vasculature are active and decisive drivers of hematogenous dissemination in breast cancer, positioning the vascular system not merely as a passive conduit for circulating tumor cells but as a dynamic regulator of metastatic organotropism. These insights highlight the clinical importance of identifying patients with underlying endothelial impairment, as this subgroup may carry a higher risk of metastatic progression. Targeted approaches that restore vascular stability, normalize tumor angiogenesis, and correct metabolic and inflammatory disturbances hold significant potential to reduce hematogenous spread and improve OS and DFS outcomes.

Furthermore, dedicated studies are needed to evaluate how AH, T2D, and ACVD influence metastatic progression. Clarifying the molecular mechanisms and signaling pathways through which these comorbidities exacerbate metastasis—particularly by promoting endothelial dysfunction—should be a priority for future research. Future directions focus on integrating vascular-targeted agents, anti-inflammatory therapy, and stabilizing the endothelial barrier through metabolic control to modulate cancer angiogenesis and to reduce metastatic spread of cancer cells. This multidisciplinary approach could decrease the rate of metastatic incidence and mortality while enhancing the effectiveness of systemic cancer treatment.

## Data Availability

No datasets were generated or analysed during the current study.

## Abbreviations

BC: Breast cancer
EC: Endothelial cells
ECM: Extracellular matrix
EMT: Epithelial–mesenchymal transition
MET: Mesenchymal–epithelial transition
MMP: Matrix metalloproteinase
IL-6: Interleukin-6
TGF: Tumor growth factor
VEGF: Vascular-endothelial growth factor
TME: Tumor microenvironment
EC: Endothelial cell
TEM: Transendothelial migration
VM: Vascular mimicry
HER2/neu: Human epidermal growth factor receptor 2
TNBC: Triple negative breast cancer
EphA2: Ephrin type receptor A 2
HIF-1α: Hypoxia inducible factor alpha
CTC: Circulating tumor cell
FSS: Fluid shear stress
PMN: Premetastatic niche
TNF-α: Tumor necrosis factor alpha
CD44: Cluster differentiation 44
MUC1: Mucin 1
VCAM-1: Vascular-cellular adhesive molecule
ICAM-1: Intercellular adhesive molecule
MVD: Microvessels density
AAT: Anti-angiogenic therapy
CD 34: Cluster differentiation 34
PR: Progesterone receptor
DNA: Deoxyribonucleic acid
CVD: Cardiovascular disease
DM: Diabetes mellitus
T1D: Type 1 diabetes
T2D: Type 2 diabetes
AH: Arterial hypertension
NO: Nitric oxide
ROS: Reactive oxygen species
eNOS: Endothelial NO synthase
RAAS: Renin-angiotensin-aldosterone system
ER: Estrogen receptor
NET: Neutrophil extracellular traps
DFS: Disease-free survival
MFS: Metastases-free survival
OS: Overall survival
WHR: Waist-to-hip ratio
NSAID: Non-steroidal anti-inflammatory drugs
PGE2: Prostaglandin E2
ACVD: Atherosclerotic cardiovascular disease
oxLDL: Oxidized low-density lipoprotein
LOX-1: Lectin-like oxidized low-density lipoprotein receptor-1
CD36: Cluster of differentiation 36
CD162: Cluster of differentiation 162
PET-CT: Positron emission tomography
